# 2P-FLIM unveils time-dependent metabolic shifts during osteogenic differentiation with a key role of lactate to fuel osteogenesis via glutaminolysis identified

**DOI:** 10.1186/s13287-023-03606-y

**Published:** 2023-12-12

**Authors:** Nuno G. B. Neto, Meenakshi Suku, David A. Hoey, Michael G. Monaghan

**Affiliations:** 1 Department of Mechanical, Manufacturing and Biomedical Engineering, Trinity College Dublin, Parsons Building, Dublin 2, Ireland; 2 CURAM SFI Research Centre for Medical Devices, National University of Ireland, Galway, Ireland; 3 Advanced Materials for Bioengineering Research (AMBER), Centre, Trinity College Dublin and Royal College of Surgeons in Ireland, Dublin, Ireland; 4 Trinity Centre for Biomedical Engineering, Trinity College Dublin, Dublin, Ireland

**Keywords:** Osteogenesis, Glutaminolysis, Metabolism, Mesenchymal stem cells, Tissue engineering, Imaging, Non-invasive characterization, Differentiation

## Abstract

**Background:**

Human mesenchymal stem cells (hMSCs) utilize discrete biosynthetic pathways to self-renew and differentiate into specific cell lineages, with undifferentiated hMSCs harbouring reliance on glycolysis and hMSCs differentiating towards an osteogenic phenotype relying on oxidative phosphorylation as an energy source.

**Methods:**

In this study, the osteogenic differentiation of hMSCs was assessed and classified over 14 days using a non-invasive live-cell imaging modality—two-photon fluorescence lifetime imaging microscopy (2P-FLIM). This technique images and measures NADH fluorescence from which cellular metabolism is inferred.

**Results:**

During osteogenesis, we observe a higher dependence on oxidative phosphorylation (OxPhos) for cellular energy, concomitant with an increased reliance on anabolic pathways. Guided by these non-invasive observations, we validated this metabolic profile using qPCR and extracellular metabolite analysis and observed a higher reliance on glutaminolysis in the earlier time points of osteogenic differentiation. Based on the results obtained, we sought to promote glutaminolysis further by using lactate, to improve the osteogenic potential of hMSCs. Higher levels of mineral deposition and osteogenic gene expression were achieved when treating hMSCs with lactate, in addition to an upregulation of lactate metabolism and transmembrane cellular lactate transporters. To further clarify the interplay between glutaminolysis and lactate metabolism in osteogenic differentiation, we blocked these pathways using BPTES and α-CHC respectively. A reduction in mineralization was found after treatment with BPTES and α-CHC, demonstrating the reliance of hMSC osteogenesis on glutaminolysis and lactate metabolism.

**Conclusion:**

In summary, we demonstrate that the osteogenic differentiation of hMSCs has a temporal metabolic profile and shift that is observed as early as day 3 of cell culture using 2P-FLIM. Furthermore, extracellular lactate is shown as an essential metabolite and metabolic fuel to ensure efficient osteogenic differentiation and as a signalling molecule to promote glutaminolysis. These findings have significant impact in the use of 2P-FLIM to discover potent approaches towards bone tissue engineering in vitro and in vivo by engaging directly with metabolite-driven osteogenesis.

**Graphical Abstract:**

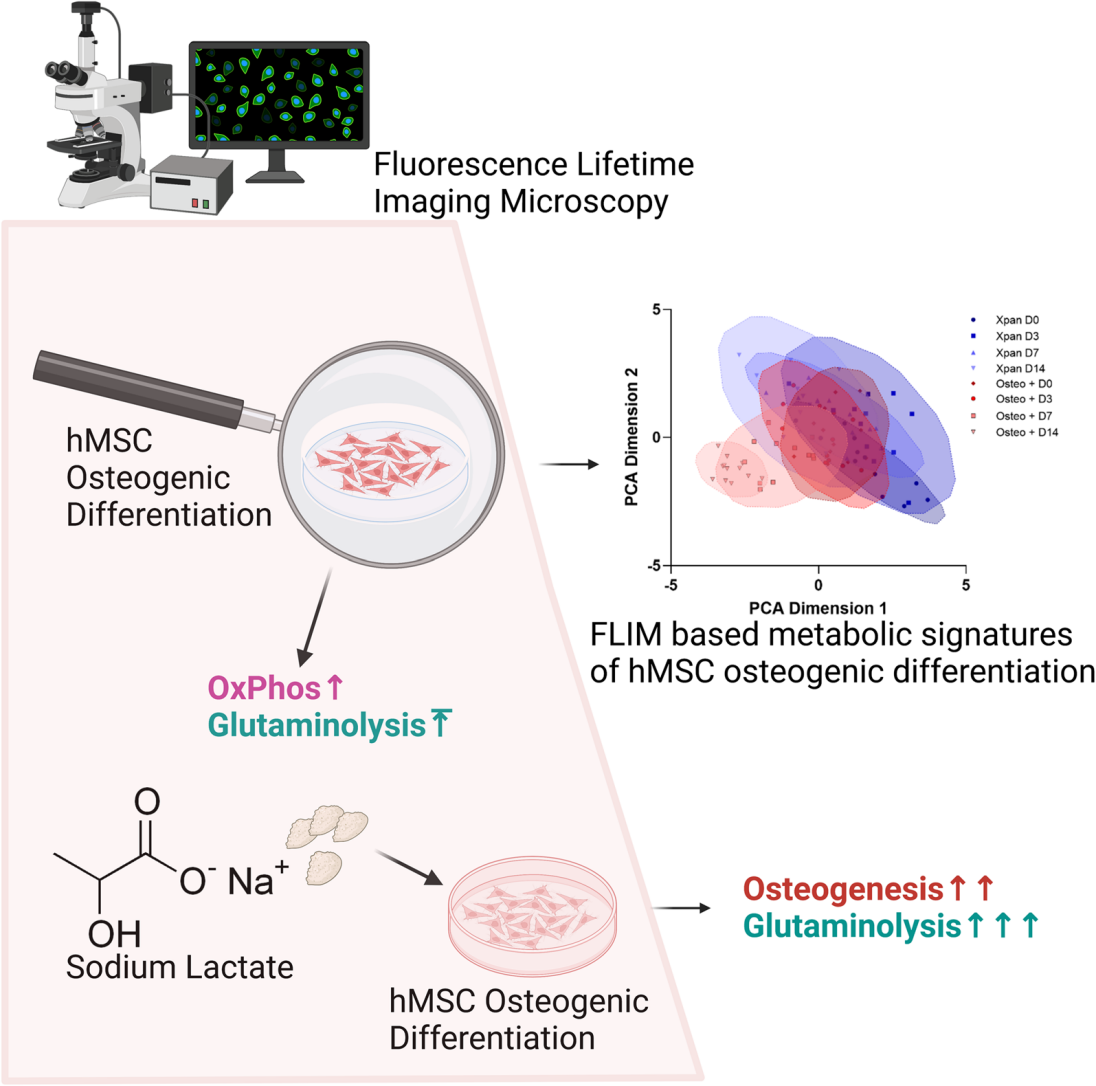

**Supplementary Information:**

The online version contains supplementary material available at 10.1186/s13287-023-03606-y.

## Introduction

Differentiation of human mesenchymal stem cells (hMSCs) towards an osteogenic, adipogenic or chondrogenic lineage is accompanied by changes in phenotype, gene expression, protein secretion and cellular metabolism that occur in a time-dependent manner. It is generally known that stromal cells rely on glycolysis for energy when undifferentiated [1]. This dependence on glycolysis facilitates a quick turnover of energy while producing biomolecules required for cellular proliferation [2]. hMSCs continue to utilize glycolysis as the main source of energy during chondrogenic differentiation [3]. However, there is a metabolic switch to oxidative phosphorylation (OxPhos) during hMSC differentiation towards adipogenic or osteogenic lineages [4]. In addition, it has been shown that an increase in oxygen consumption, mitochondrial activity and glutamine metabolism are important for osteogenic differentiation of hMSCs [5], which highlights metabolic pathways as a mechanism to drive a desired cell phenotype.

Multiple tools exist to understand cellular metabolism. Particularly, two-photon fluorescence lifetime imaging (2P-FLIM) is a powerful technique for non-invasive probing and monitoring of cellular metabolism. This technique is based on the fluorescence properties of nicotinamide adenine dinucleotide (NAD(P)H) and flavin adenine dinucleotide (FAD). NAD(P)H fluorescence lifetime consists of short and long lifetime components corresponding, respectively, to free and protein-bound forms [6]. NAD(P)H is an important metabolic co-factor that drives ATP production and other anaplerotic metabolic pathways. NAD(P)H drives glycolysis in the cytoplasm and oxidative phosphorylation (OxPhos) in the mitochondria. Therefore, longer fluorescence lifetimes are associated with higher ratios of protein-bound NAD(P)H revealing a higher dependence of OxPhos as a source of ATP. NAD(P)H fluorescence signal, especially protein-bound NAD(P)H (*τ*_1_), can be used to infer quantitatively in NADPH concentrations [7]. NADPH has an important role in lipid, amino acid and nucleotide biosynthetic pathways as well as reactive oxygen species (ROS) protection [8]. FAD^+^ is also an important metabolic co-factor for OxPhos. Specifically, FAD^+^ is a proton acceptor during the Krebs cycle and a proton donor on the electron transport chain (ETC) [9]. The ratios of the fluorescence intensities of FAD and NAD(P)H (FAD/NAD(P)H) are referred to as optical redox ratio and it can be used to estimate the metabolic profile of cells or tissues [10]. 2P-FLIM has been used as a technique to investigate cellular metabolism in a variety of cells, tissues and organoids [11, 12] and directly in vivo [13]. Metabolic probing of hMSCs during differentiation using 2P-FLIM has been previously reported. Meleshina et al. [14] applied 2P-FLIM to monitor hMSCs metabolic profile while in osteogenic differentiation culture for 21 days with an increase of OxPhos observed as early as day 7 of cell culture. After 2P-FLIM imaging, it is still possible to probe the sample further via metabolic, biochemical or histological validation [15]. In addition, machine learning models to the data analysis can allow additional understanding of the impact of metabolic treatments [16].

**Fig. 1:**
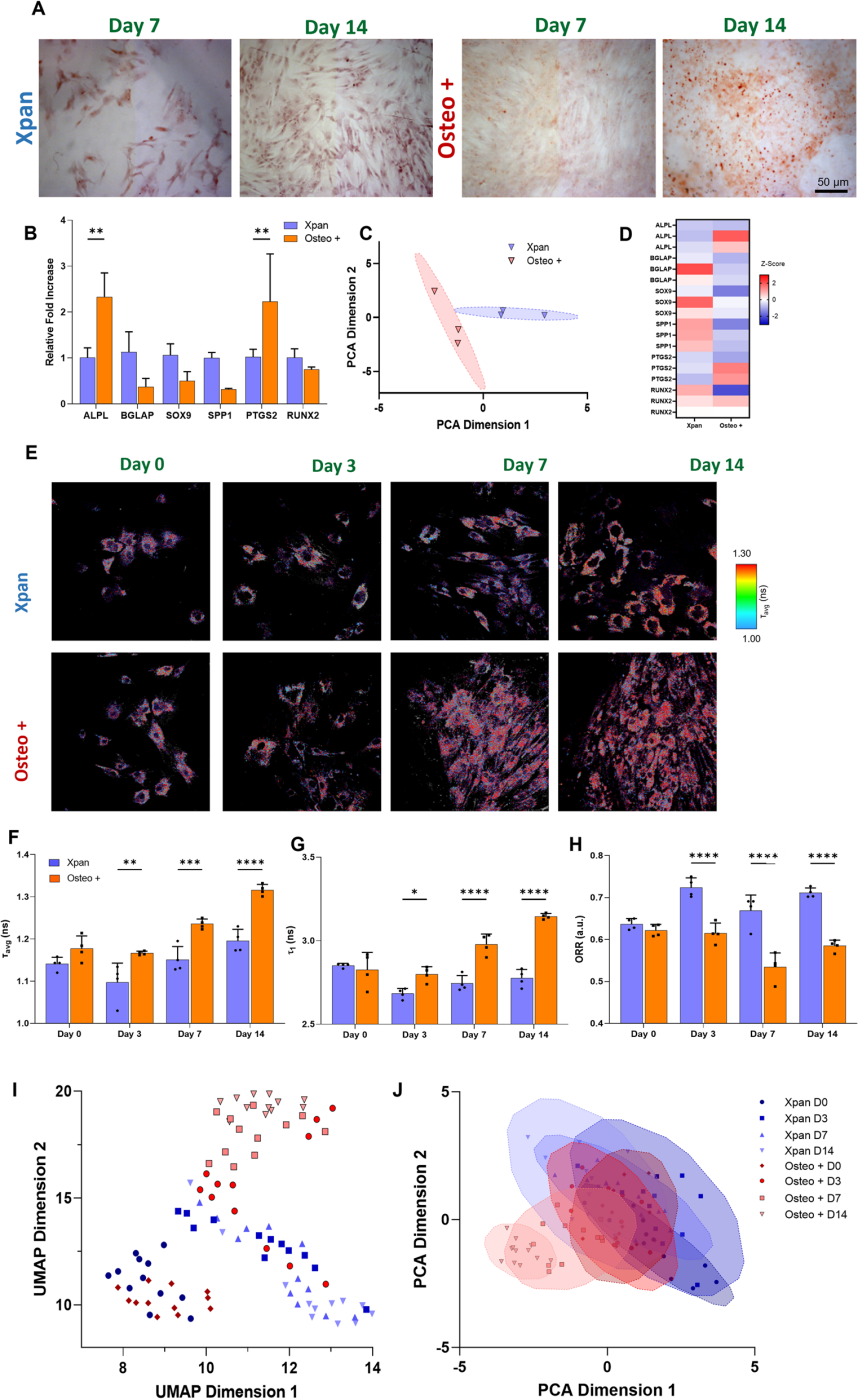
Validation of osteogenic differentiation of hMSCs incubated in Osteo + or Xpan cell culture medium for 14 days. **A** Alizarin red staining of hMSCs after 14 days culture in Osteo + or Xpan medium. **B** hMSCs gene expression of osteogenic markers after 14 days of cell culture. **C** PCA distribution and of hMSCs osteogenic gene expression after 14 days of cell culture. **D** *Z*-score heatmap of hMSCs gene expression of osteogenic gene markers after 14 days of cell culture. **E** 2P-FLIM imaging of hMSCs at day 0, 3, 7 and 14 of cell culture incubation. **F** NAD(P)H *τ*_avg_ measurement of hMSCs at day 0, 3, 7 and 14 of cell culture. **G** NAD(P)H protein-bound lifetime *τ*_1_ at day 0, 3, 7 and 14 of cell culture. **H** Optical redox ratio (ORR) of hMSCs at day 0, 3, 7 and 14 of incubation in cell culture medium. **I** UMAP distribution of 2P-FLIM NAD(P)H and FAD^+^ fluorescence lifetimes and intensity. **J** PCA distribution of 2P-FLIM NAD(P)H and FAD^+^ fluorescence lifetimes and intensity. *p-value ≤ 0.05, **p-value ≤ 0.01, ***p-values ≤ 0.001, ****p-value ≤ 0.0001 *N* ≥ 3

In this study, we first profile and classify the osteogenic differentiation of hMSCs using 2P-FLIM and validate the metabolic profiles observed using extracellular metabolite quantitation and gene expression analysis (Fig. 1). Following this, we assess the potential of interrogating metabolism to promote osteogenic differentiation of hMSCs and monitor this using conventional assays and non-invasively classify using 2P-FLIM. Glutaminolysis is an extensively documented metabolic pathway, which has also been shown to be crucial in ensuring adequate osteogenic differentiation of hMSCs [17, 18]. We demonstrate that upregulating glutaminolysis via lactate supplementation can drive osteogenic differentiation. Finally, using 2P-FLIM, we show that blocking glutaminolysis and lactate metabolism can severely impair osteogenic differentiation, demonstrating the impact and efficiency of metabolite-driven osteogenic differentiation of hMSCs to improve bone regeneration.

## Methods

### Cell culture

Human mesenchymal stromal cells (hMSCs) were purchased from Lonza^®^ were cultured at passage 3–4 in expansion media (Xpan) prepared with Dulbecco’s modified Eagle’s medium (DMEM), 1000 mg/L low glucose (Sigma-Aldrich) containing 10% foetal bovine serum (FBS) (Gibco^®^ by Life Technologies) and 2% Penicillin Streptomycin (Pen-Strep) (Sigma-Aldrich) at 37 °C with 5% CO_2_. To initiate osteogenic differentiation, osteogenic media (Osteo +) was prepared by adding 0.2% (v/v) dexamethasone, 1% (v/v) β-glycerol phosphate and 0.029% (v/v) ascorbic acid as supplements to Xpan media.

For lactate supplementation of Osteo + media, 7.5 mM of lactate was used as a sodium salt, to avoid changes in extracellular pH (Osteo + Lact). For metabolic inhibition studies, either 5 mM of α-CHC or 10 μM BPTES was added to Osteo + media.

### Two-photon fluorescence lifetime imaging microscopy (2P-FLIM)

2P-FLIM was performed on hMSCs (at passage 3–4) seeded in 35-mm petri dishes. 2P-FLIM was achieved using a custom upright (Olympus BX61WI) laser multiphoton microscopy system equipped with a pulsed (80 MHz) titanium: sapphire laser (Chameleon Ultra, Coherent^®^, USA), water immersion 25 × objective (Olympus, 1.05NA) and temperature-controlled stage at 37 °C. Two photon excitation of NAD(P)H and FAD^+^ fluorescence was performed at the excitation wavelength of 760 and 800 nm, respectively. A 458/64 nm and 520/35 nm band-pass filter were used to isolate the NAD(P)H and FAD^+^ fluorescence emissions based on their emission spectrum.

512 × 512 pixel images were acquired with a pixel dwell time of 3.81 μs and 30 s collection time. A PicoHarp 300 TCSPC system operating in the time-tagged mode coupled with a photomultiplier detector assembly (PMA) hybrid detector (PicoQuanT GmbH, Germany) was used for fluorescence decay measurements, yielding 256 time bins per pixel. TCSPC requires a defined “start”, provided by the electronics steering the laser pulse or a photodiode, and a defined “stop” signal, realized by detection with single-photon-sensitive detectors. The measuring of this time delay is repeated many times to account for the statistical variance of the fluorophore’s emission. For more detailed information, the reader is referred elsewhere [19]. Fluorescence lifetime images with their associated decay curves for NAD(P)H were obtained, with a minimum of 1 × 10^6^ photons peak, and region of interest (ROI) analysis of the total cells present on the image was performed in order to remove any background artefact. The decay curved was generated and fitted with a double-exponential decay without including the instrument response function (IRF) (Eq. (1)).

1 $$I\left( t \right) = I\left( 0 \right)\left[ {\alpha_{1} e^{{\frac{ - t}{{\tau_{1} }}}} + \alpha_{2} e^{{\frac{ - t}{{\tau_{2} }}}} } \right] + C$$

*I*(*t*) represents the fluorescence intensity measured at time t after laser excitation; *α*_1_ and *α*_2_ represent the fraction of the overall signal proportion of a short and long component lifetime, respectively; *τ*_1_ and *τ*_2_ are the long and short lifetime components, respectively; and C corresponds to background light. Chi-squared statistical test was used to evaluate the goodness of multi-exponential fit to the raw fluorescence decay data. In this study, all the fluorescence lifetime fitting values with χ^2^ < 1.3 were considered as “good” fits. For NAD(P)H, the double exponential decay was used to differentiate between the protein-bound (*τ*_1_) and free (*τ*_2_) NAD(P)H. The average fluorescence lifetime was calculated using Eq. (2).

2 $$\tau_{avg} = \frac{{\left( {\tau_{1} \times \alpha_{1} + \tau_{2} \times \alpha_{2} } \right)}}{{(\alpha_{1} + \alpha_{2} )}}$$

Intensity-based images of NAD(P)H and FAD^+^ were acquired, and their ratio was calculated using Eq. (3) to obtain the optical redox ratio (ORR).

3 $${\text{ORR}} = \frac{{{\text{FAD}}^{ + } }}{{{\text{NAD}}\left( {\text{P}} \right){\text{H}}}}$$

### qPCR analysis

hMSCs were lysed and re-suspended in 1 ml of TRIzol, and briefly vortexed, and 200 μL of chloroform was added. Samples were centrifuged at 12,000*g* for 15 min at 4 °C to achieve phase separation. The aqueous phase was separated to a fresh RNAse free microtube. RNA precipitation was performed by adding 2 μL of glycol-blue and 500 μL of isopropanol. RNA pellets were washed using 1 mL of 70% of ethanol. RNA pellets were re-suspended in 20 μL of RNAse free water. RNA yield and purity were measured using a NanoDrop™. To estimate total RNA concentration, absorbance values obtained at 260 nm were obtained. 260/280 and 260/230 absorbance ratios were calculated to estimate RNA purity. A 260/280 ratio of ~ 1.8 and a 260/230 ratio of ~ 1.8 were deemed acceptable for RNA purity. cDNA transcription was performed with high-capacity cDNA reverse transcription kit from Applied Biosystems™ according to manufactures instructions. A master mix solution was prepared by adding RNase inhibitor, Multiscribe™ reverse transcriptase enzyme, RT^®^ buffer, random primers and 100 mM of dNTP Mix. RNA samples were added to the mastermix to achieve a reaction volume of 20 μL. A thermal cycle was performed with a heating step of 25 °C for 10 min, 37 °C for 120 min, followed by 85 °C for 5 min. A 100% conversion rate was assumed to determine cDNA concentration.

Gene expression was analysed using a custom PrimePCR^®^ designed plate using SYBR^®^ Green primers according to manufactures guidelines. Here, 10 μL of Universal SYBR^®^ Green Supermix is added to each well of the 96-well plate to reconstitute the dried PCR primers. 4 μL of cDNA samples was added to each well, and nuclease-free water was used to achieve 20 μL of reaction volume. All primers were purchased from Bio-Rad laboratories. Accession numbers, Unique Assay ID, Amplicon Length, Gene name and symbol are present in Additional file 1: Table S1. Primer sequences remain Bio-Rad laboratories proprietary information and are not available. PCR primers specificity are verified and guaranteed by the manufacturer. qPCR was performed on a fastPCR 96-well plate instrument (Applied Biosystems) to obtain comparative ΔΔCt values. Gene expression was compared between samples using the 2^−ΔΔCt^ method. qPCR data were normalized to β-actin gene expression.

*Z*-score heatmaps of gene expression values were generated by calculating the *z*-score of each sample and plotted in a heatmap. *Z*-score values are obtained using Eq. 4:

4 $$z = \frac{x - \mu }{\sigma }$$

The *z*-score (*z*) is calculated using $x$ the observed value, *μ* the mean value of the sample and σ the standard deviation of the sample.

### Metabolite analysis

#### Glucose

For metabolite analysis, cell culture medium was collected at 2, 5, 8 and 11 days after being in contact with cell cultures. For extracellular measurement of glucose, the ChromaDazzle™ Glucose Assay kit from AssayGenie was used. Here, glucose standards were prepared by dissolving glucose stock solution 300 mg/dL in distilled water with concentrations ranging from 0 to 300 mg/dL to prepare a standard curve. Cell culture medium samples and standards were diluted 1/100 in o-toluidine reagent from AssayGenie. Samples were incubated in a boiling water bath for 8 min and cooled down in ice for 4 min. 200 μL was added to clear-bottomed 96-well plates, and absorbance values were measured in a microplate reader at 630 nm.

#### Lactate

Extracellular measurements of lactate were taken using an l-lactic acid colorimetric assay by AssayGenie. Here, lactate standards were obtained by sequentially diluting a 10 mM/L lactate standard to obtain a standard curve with the concentration ranging from 0 to 7 mM/L. Samples and standards were added directly to 96-well plates and diluted 60 × in chromogenic solution. For samples with increased exogenous lactate, 12 × dilutions were performed. Samples were incubated at 37 °C for 5 min and read in a microplate reader at 530 nm.

#### Glutamine

Extracellular measurements of glutamine were taken using ChromaDazzle™ Glutamine Assay kit from AssayGenie. This kit is based on the hydrolysis of glutamine to glutamate and formation of a colorimetric product. First, a standard curve was calculated by diluting glutamine stock solution 100 mM in distilled water. Standards with concentrations from 0 to 2 mM were obtained. Enzymes A qnd B from the kit were reconstituted according to manufacturer’s instructions. Extracellular culture medium and standards were added to clear-bottomed 96-well plates and diluted 10 × in assay solution containing enzymes. The enzymatic reaction was stopped at 40 min at room temperature with a stop reagent, and absorbance values were measured in a plate reader at 550 nm.

In this study, there is an interest in measuring glucose and glutamine consumption. Therefore, to calculate metabolite concentration Eq. 5 was used. For glucose initial concentration was assumed 1000 mg/L and 2.0 mM for glutamine concentration according to manufactures formulations. Extracellular lactate concentration measurements are presented directly without using Eq. 4. In addition, for lactate measurements in lactate supplemented osteogenic medium were corrected by taking in account the amount of lactate added exogenously.

5 $$\Delta \left[ {{\text{metabolite}}} \right]_{{\text{medium }}} = \left[ {{\text{metabolite}}} \right]_{{{\text{initial}}}} - \left[ {{\text{metabolite}}} \right]_{{{\text{final}}}}$$

### PCA and UMAP

Uniform manifold approximate and projection (UMAP) and principal component analysis (PCA) were used as data visualization tools to visualize clustering within 2P-FLIM data variables (Python). For this analysis, all images acquired (at least 3 per sample and condition) were used as plot points and 2P-FLIM variables or gene expression data were used as variables. Covariance error ellipses with confidence values of 99% were calculated and plotted in PCA graphs. Mahalanobis distance, two-sample Hotelling’s *T*^2^ statistic, *F*-value, critical *F*-Value and *p*-values were calculated to ensure statistical significant separation of PCA clusters (Additional file 1: Table S2).

### Alizarin red staining (ARS)

hMSCs were fixed with 4% paraformaldehyde (PFA), and alizarin red staining was performed for 30 min with a solution of 1% alizarin red in distilled water and imaged with a 10 × objective using an Olympus BX41TF bright-field microscope. Alizarin red molecules bind to calcium molecules and generate a birefringent insoluble red complex. Alizarin red stained cells were dissolved for 30 min in 10% (v/v) acetic acid. Cells were lifted with a cell scrapper and transferred to microtubes and heated until 85 °C for 10 min. The tubes were centrifuge at 20,000 g for 15 min, and 10% (v/v) ammonium hydroxide was added. Samples were pipetted in triplicate on a 96-well plate, and absorbance was measured in a plate reader at 405 nm.

### DNA quantification

For sample lysis, hMSCs were rinsed twice using cold phosphate-buffered saline solution (PBS) and were digested using a lysis buffer containing 0.2% v/v Triton X100, 10 mM Tris pH 8, 1 mM EDTA and DNAse free water. Afterwards, the cells suspended in lysis buffer were transferred to microtubes and sonicated for 60 s to ensure complete cellular lysis.

DNA quantification was performed using a PicoGreen™ biochemical assay on digested samples according to manufacturer’s protocol. Here, PicoGreen™ was diluted in 10 mM Tris–HCl and 1 mM EDTA with pH 7.5 (TE). A DNA stock solution (100 μg/mL) was dissolved in TE solution, and serial dilution from 0 to 200 ng of DNA/mL was performed to establish a calibration curve. Afterwards, 10 μL of each standard and sample were added in triplicate to a black flat-bottomed 96-well plate and further 190 μL of diluted PicoGreen™ in TE solution was added. Fluorescence emission was read at 520 nm using an excitation wavelength of 480 nm in a plate reader with 0.93 s per well of dwell time.

### Statistics

Statistical analysis was performed using GraphPad Prism 9 (GraphPad Software, USA). Where appropriate, one-way analysis of variance (ANOVA) or two-way ANOVA were used as statistical tests followed by Tukey’s multiple comparison. Results are presented as mean ± standard deviation, and differences are considered as statistically significant for *p*-value ≤ 0.05.

## Results

### Osteogenic differentiation involves oxidative phosphorylation in a time-dependent manner

hMSCs were cultured in expansion media (Xpan) and Xpan supplemented with the osteogenic factors 0.2% (v/v) dexamethasone, 1% (v/v) β-glycerol phosphate and 0.029% (v/v) ascorbic acid (Osteo + media) for 14 days. On day 7 and day 14 of culture, hMSCs were fixed using paraformaldehyde (PFA) and stained with alizarin red to verify mineral deposition and osteogenic differentiation (Fig. 1A). As expected, after 14 days of incubation a noticeably higher mineral deposition in Osteo + medium conditions was observed, associated with increased osteogenic differentiation (Fig. 1A, Additional file 1: Fig. S1). After 14 days of culture in either Xpan or Osteo + media, the osteogenic gene expression of hMSCs was quantified using qPCR (Fig. 1B, C, D). A statistically significant increase in the gene expression of alkaline phosphatase (ALPL, 2.3-fold increase) and prostaglandin-endoperoxide synthase 2 (PTGS2, 2.19-fold increase) was observed in hMSCs when cultured in Osteo + medium (Fig. 1B). The expressions of the remaining osteogenic genes were found to be not statistically significant between Osteo + and Xpan media conditions. PCA visualization of osteogenic gene expression revealed a statistically significant segregation between hMSCs incubated in Xpan or Osteo + cell culture medium with *p*-value < 0.05 and *F*-value higher than critical *F*-value (Fig. 1C, Additional file 1: Table S2). A *z*-score heatmap based on osteogenic gene expression shows similar results with a higher expression of ALPL and PTGS2 in Osteo + conditions after 14 days of incubation (Fig. 1C).

2P-FLIM of NAD(P)H and FAD^+^ revealed a time-dependent metabolic shift with hMSCs in osteogenic medium (Fig. 1E–J). A higher *τ*_avg_ is reflective of a metabolic shift towards OxPhos as a consequence of a higher proportion of longer lifetime and protein-bound NAD(P)H [6]. Also, increased *τ*_1_ lifetimes highlight higher amounts of NADPH and anabolic activity [7]. On day 0, no significant differences in *τ*_avg_, *τ*_1_ or optical redox ratio (ORR) values were observed between hMSCs cultured in Xpan or Osteo + media (Fig. 1F, G, H). On day 3 of cell culture, significantly higher (1.097 ± 0.04 ns versus 1.167 ± 0.004 ns) *τ*_avg_ and (2.683 ± 0.027 ns versus 2.800 ± 0.039 ns) *τ*_1_ values concomitant with lower (0.724 ± 0.020 versus 0.615 ± 0.021) ORR values (Fig. 1F, G, H) were observed. The trends observed on day 3 became more accentuated with longer-term cultures. NAD(P)H fluorescence *τ*_avg_ (1.167 ± 0.004 ns at day 3, versus 1.236 ± 0.010 ns at day 7 and 1.316 ± 0.011 ns at day 14) and *τ*_1_ (2.800 ± 0.039 ns at day 3, versus 2.979 ± 0.053 ns at day 7 and 3.146 ± 0.015 ns at day 14) was found to be higher in Osteo + treated cells while in Xpan conditions, and these fluorescence variables remained constant. Varying interpretations of ORR are reported in previous studies [20], and for this study, we adopt a lower ORR reflecting a higher fraction of NAD(P)H and a lower FAD^+^ associated with upregulation of Krebs cycle. An increased generation of NADH will result in a decreased ORR (Eq. 3) [21]. ORR values continued to decrease in Osteo + conditions after day 3 (0.615 ± 0.021 ns at day 3, versus 0.534 ± 0.029 ns at day 7 and 0.585 ± 0.012 ns at day 14) while hMSCs in Xpan medium had stable ORR values (Fig. 1F, G, H) which we hypothesized to be a result of an increased dependence on anabolic pathways such as glutaminolysis.

UMAP and PCA were used as exploratory data visualization tools to cluster the 2P-FLIM-derived photonic data linked to the metabolic profiles of hMSCs undergoing osteogenic differentiation at several time points during cell culture (Fig. 1I, J). All 2P-FLIM NAD(P)H and ORR variables were used to generate the UMAP and PCA plots. UMAP revealed that at later time points, such as day 7 and day 14, there is a strong separation between hMSCs cultured in either Osteo + or Xpan media (Fig. 1I). For earlier time points, there was no segregation between hMSCs cultured in both cell culture medium conditions (Fig. 1I, Additional file 1: Table S2). When applying PCA, there is a clear and statistically significant segregation of cells treated with Osteo + media at day 7 and day 14 compared with hMSCs cultured in Xpan media (Fig. 1J, Additional file 1: Table S2).

### Lactate supplementation promotes higher glutaminolysis, lactate metabolism and poly-hydrolases gene expression

**Fig. 2:**
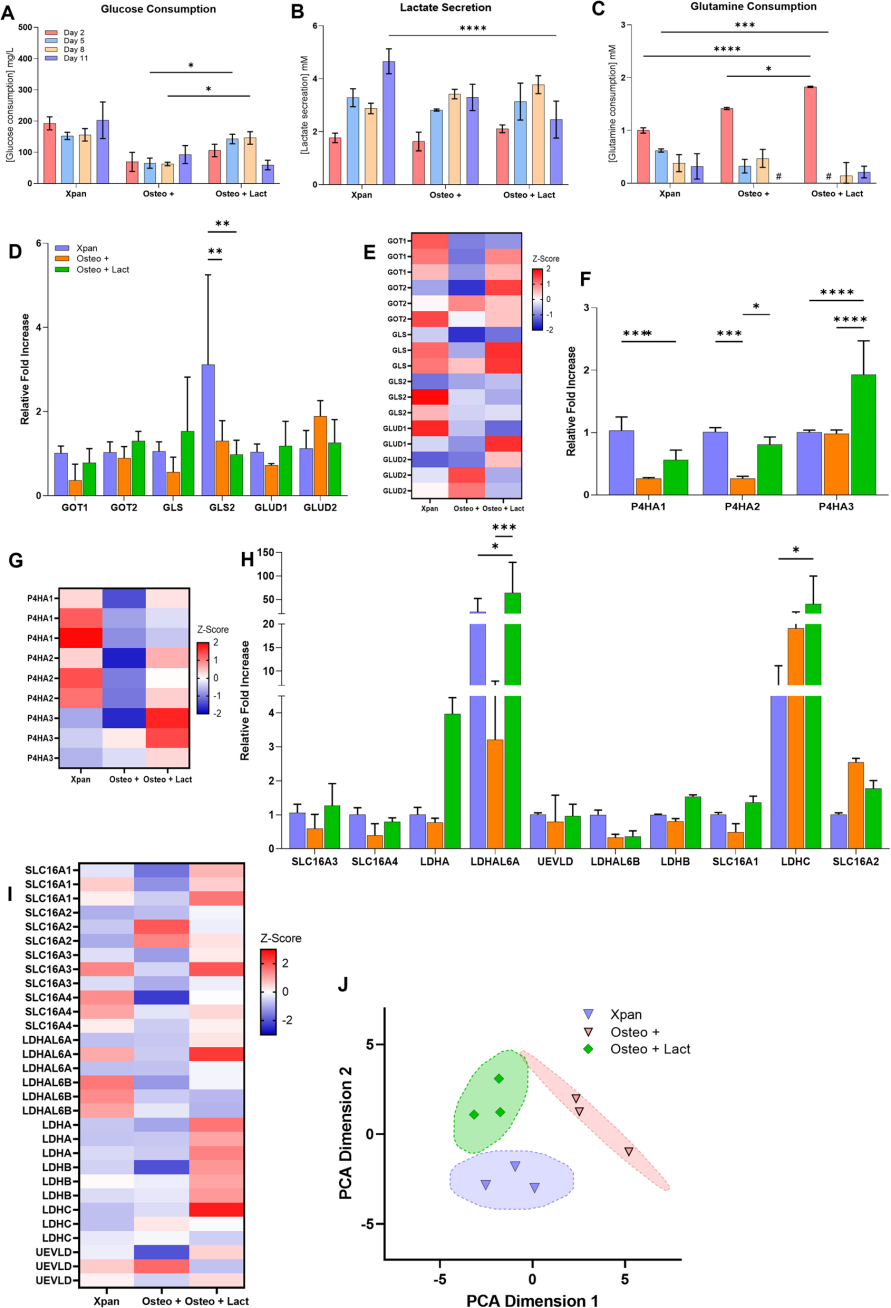
Impact of lactate supplementation of osteogenic differentiation media on the metabolites and on the expression of metabolic genes. **A** hMSCs glucose consumption at day 2, 5, 8 and 11 of cell culture. **B** Lactate secretion at day 2, 5, 8 and 11 of cell culture. **C** hMSCs glutamine consumption at day 2, 5, 8 and 11 of cell culture. **D** hMSCs relative fold variation of glutaminolysis genes. **E** *Z*-score heatmap of genes expression related to glutaminolysis. **F** hMSCs relative fold variation of poly-hydrolases genes. **G** *Z*-score heatmap of genes expression of poly-hydrolases. **H** hMSCs relative fold variation of lactate transport and metabolism genes. **I** *Z*-score heatmap of genes expression related to lactate transport and metabolism **J** PCA distributions of cell culture conditions at day 14 based on glutaminolysis, lactate metabolism and poly-hydrolase gene expression. ^#^no glutamine consumption, *p-value ≤ 0.05, **p-value ≤ 0.01, ***p-values ≤ 0.001, ****p-value ≤ 0.0001 *N* ≥ 3

After detecting, using 2P-FLIM that osteogenic differentiation of hMSCs potentially promoted a temporal metabolic shift towards OxPhos and increased dependence on anabolic pathways such as glutaminolysis (as captured in Fig. 1), we increased dependence of glutaminolysis and anabolic pathways by adding exogenous sodium lactate to osteogenic medium [22]. Cell culture media was collected during media exchanges at days 2, 5, 8 and 11 from which glucose, lactate production and glutamine concentrations were measured using colorimetric assays (Fig. 2A, B, C). The purpose of this investigation was twofold: to clarify the utilization of hMSCs undergoing osteogenesis on glutaminolysis and to drive this further using exogenous lactate.

Regarding glucose consumption, a statistically significant increase was observed (2.42-fold increase) when cells were cultured in Xpan medium at all time points (Fig. 2A). On day 5 and 8, glucose consumption of hMSCs in lactate supplemented osteogenic medium was found to be significantly higher (2.26-fold change) compared to those with Osteo + medium (Fig. 2A). On day 11, lactate supplementation was found to promote a decrease in the glucose consumption of hMSCs (Fig. 2A). Lactate supplementation promoted a statistically significantly lower glucose consumption compared to Xpan medium at early time points. Additionally an increasing temporal trend in lactate secretion was noted in both Xpan and Osteo + treated hMSCs during the incubation period. For lactate secretion measurements of lactate supplemented osteogenic medium, the contribution of exogenous lactate was taken into account and the values were corrected. On day 11, there was a statistically significant higher lactate secretion in hMSCs cultured in Xpan medium (4.660 ± 0.384 mM) compared with hMSCs in Osteo + (3.295 ± 0.406 mM) culture conditions (Fig. 2B). Noteably, a significantly higher glutamine consumption was found on day 2 of hMSCs in Osteo + (1.417 ± 0.017 mM) compared to Xpan (1.000 ± 0.041 mM) cell culture conditions. For the remaining time points, there was a decreasing trend of glutamine consumption for both cell culture media conditions (0.01-fold for Osteo + and 0.32-fold for Xpan) (Fig. 2C). When hMSCs were incubated in Osteo + Lact medium, a higher consumption of glutamine was found on day 2 (1.827 ± 0.009 mM) compared to other cell culture media (1.417 ± 0.017 for Osteo + and 1.000 ± 0.041 for Xpan) (Fig. 2C). For the remaining time points, a sharp decrease in glutamine consumption was observed for lactate supplemented osteogenic medium (Fig. 2C).

Next, we sought to evaluate glutaminolysis, lactate metabolism and poly-hydrolase gene expression, to further establish the impact of lactate supplementation on these metabolic pathways. hMSCs were cultured for 14 days in Xpan, Osteo +  and Osteo + Lact culture conditions. Afterwards, the expression of genes related to glutaminolysis, poly-hydrolases, lactate metabolism and lactate transport was measured using qPCR. Osteo + Lact cell culture medium promoted an increase in expression of glutaminolysis-related genes such as GOT1 (2.17-fold), GOT2 (1.46-fold), GLS (2.73-fold) and GLUD1 (1.64-fold) when compared with non-supplemented osteogenic medium (Fig. 2D, E). Osteo + Lact induced an increase expression of P4HA1 (2.15-fold), P4HA2 (3.12-fold) and specially P4HA3 (1.97-fold) poly-hydrolases when compared with Osteo + medium (Fig. 2F, G). Furthermore, Osteo + Lact medium also promoted the increase expression of lactate transporter genes such as SLC16A1 (2.78-fold), SLC16A3 (2.15-fold), SLC16A4 (2.00-fold) and lactate metabolic genes LDHAL6A (19.89-fold), LDHAL6B (1.09-fold), LDHA (5.16-fold), LDHB (1.89-fold), LDHC (2.12-fold) (Fig. 2H, I). Finally, PCA distribution of cell culture conditions calculated based on the expression of metabolism-related genes showed a statistically significant segregation between Osteo + , Osteo + Lact and Xpan cell culture conditions with *p*-value < 0.05 and calculated *F*-value higher than critical *F*-value (Fig. 2J).

### Lactate supplementation promotes higher mineral deposition and impacts metabolism

**Fig. 3:**
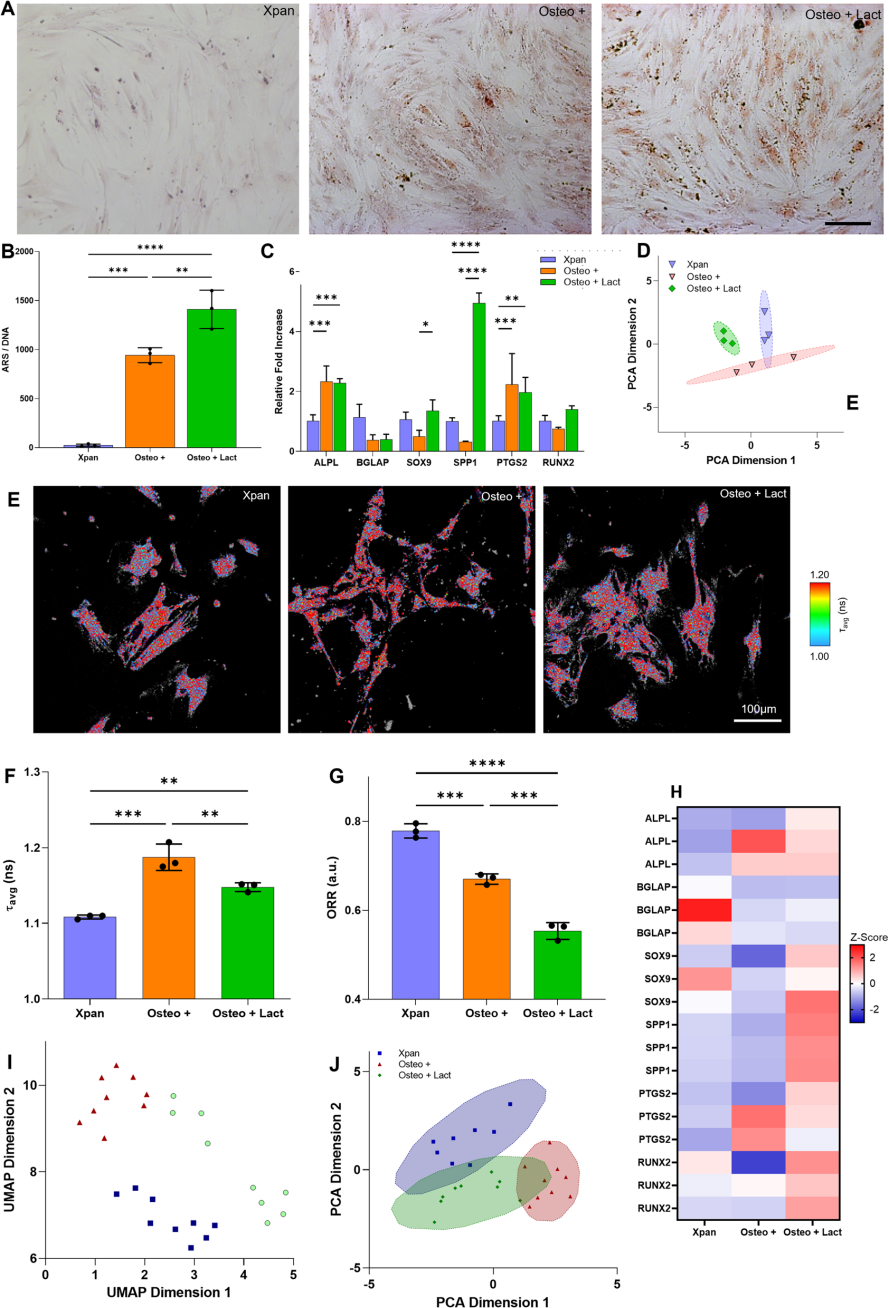
Impact of lactate supplementation of osteogenic differentiation media. **A** Alizarin red staining of hMSCs after 14 days in contact with Osteo + , Osteo + Lact or Xpan medium. **B** Quantification of Alizarin red staining per amount of DNA after 14 days of hMSCs in Xpan, Osteo + and Osteo + Lact conditions. **C** hMSCs gene expression of osteogenic markers after 14 days of cell culture. **D** PCA distribution of hMSCs osteogenic gene expression after 14 days of cell culture. **E** 2P-FLIM imaging of hMSCs at day 14 of cell culture incubation. **F** NAD(P)H protein-bound lifetime *τ*_1_ at day 14 of cell culture. **G** Optical redox ratio (ORR) of hMSCs at day 0, 3, 7 and 14 of incubation in cell culture medium. **H** *Z*-score heatmap of hMSCs gene expression of osteogenic gene markers after 14 days of cell culture. **I** UMAP distribution of 2P-FLIM NAD(P)H and FAD^+^ fluorescence lifetimes and intensity. **J** PCA distribution of 2P-FLIM NAD(P)H and FAD^+^ fluorescence lifetimes and intensity. *p-value ≤ 0.05, **p-value ≤ 0.01, ***p-values ≤ 0.001, ****p-value ≤ 0.0001 *N* ≥ 3

Lactate supplementation of osteogenic medium (at a concentration of 7.5 mM) yielded higher levels of mineral deposition after 14 days of culture with a higher amount of alizarin red staining when compared with Osteo + medium (Fig. 3A, B). Osteogenic gene expression was assessed by qPCR in hMSCs cultured in Osteo + , Xpan and lactate supplemented osteogenic medium (Fig. 3C, D, H). We observed a statistically significant increase in SRY-Box Transcription Factor 9 (SOX9) (2.7-fold increase) and Osteopontin (SPP1) gene expression (15.47-fold increase) in hMSCs when osteogenic medium was supplemented with lactate compared to Osteo + and Xpan cell culture medium (Fig. 3C). The gene expression RUNX2 was also found to be upregulated in lactate supplemented osteogenic medium compared with Xpan (1.39-fold increase) and Osteo + (1.87-fold increase) incubation medium (Fig. 3C). PCA distribution plot of cell culture conditions of hMSCs at day 14 shows a statistically significant segregation between hMSCs incubated in Osteo + Lact, Osteo + and Xpan based on the expression of their osteogenic genes with *p*-value < 0.05 and calculated *f*-value higher than critical *f*-value (Fig. 3D, Additional file 1: Table S2). ALPL, BGLAP, PTGS2 have similar gene expression values in hMSCs when incubated in osteogenic medium with and without lactate supplementation, as observed in the bar plot and the *z*-score heatmap (Fig. 3C, H).

2P-FLIM imaging of NAD(P)H was conducted at day 14 of cell culture (Fig. 3E). 2P-FLIM revealed a statistically significant lower *τ*_avg_ when supplementing osteogenic medium with lactate (1.148 ± 0.005 ns) compared with non-supplemented osteogenic medium (1.187 ± 0.014 ns) (Fig. 3F). However, *τ*_avg_ of Osteo + Lact (1.148 ± 0.005 ns) is statistically significantly higher compared with hMSCs incubated in Xpan medium (1.108 ± 0.002 ns) (Fig. 3F). ORR calculations showed statistically significant lower ORR values for Osteo + Lact medium (0.547 ± 0.016 ns) compared with other cell culture medium formulations (0.779 ± 0.016 ns for Xpan and 0.668 ± 0.011 ns for Osteo +) (Fig. 3G). The decrease in *τ*_avg_ and decrease in ORR ratios demonstrate a direct metabolic impact on differentiating hMSCs when cell culture medium is supplemented with lactate when compared with non-supplemented osteogenic media. A decrease in *τ*_avg_ is associated with increased free NAD(P)H and a decrease in ORR is also related to an increase in the NAD(P)H fluorescence intensity. The increase in NAD(P)H can be a direct result of increased glutaminolysis resulting in NADH regeneration in the conversion of glutamate to alpha-ketoglutarate as well as with NADH production by reconversion of lactate to pyruvate [23]. UMAP distribution analysis of 2P-FLIM results shows a separation between Xpan, Osteo + , Osteo + Lact (Fig. 3I). PCA shows a slight overlap of Osteo + Lact with Osteo + and Xpan medium conditions. Nonetheless, statistical analysis of PCA clustering revealed a statistically significant segregation with p-value < 0.05 and calculated *f*-value higher than critical *f*-value (Fig. 3J, Additional file 1: Table S2).

### Blocking glutaminolysis and lactate absorption impacts osteogenic differentiation

**Fig. 4:**
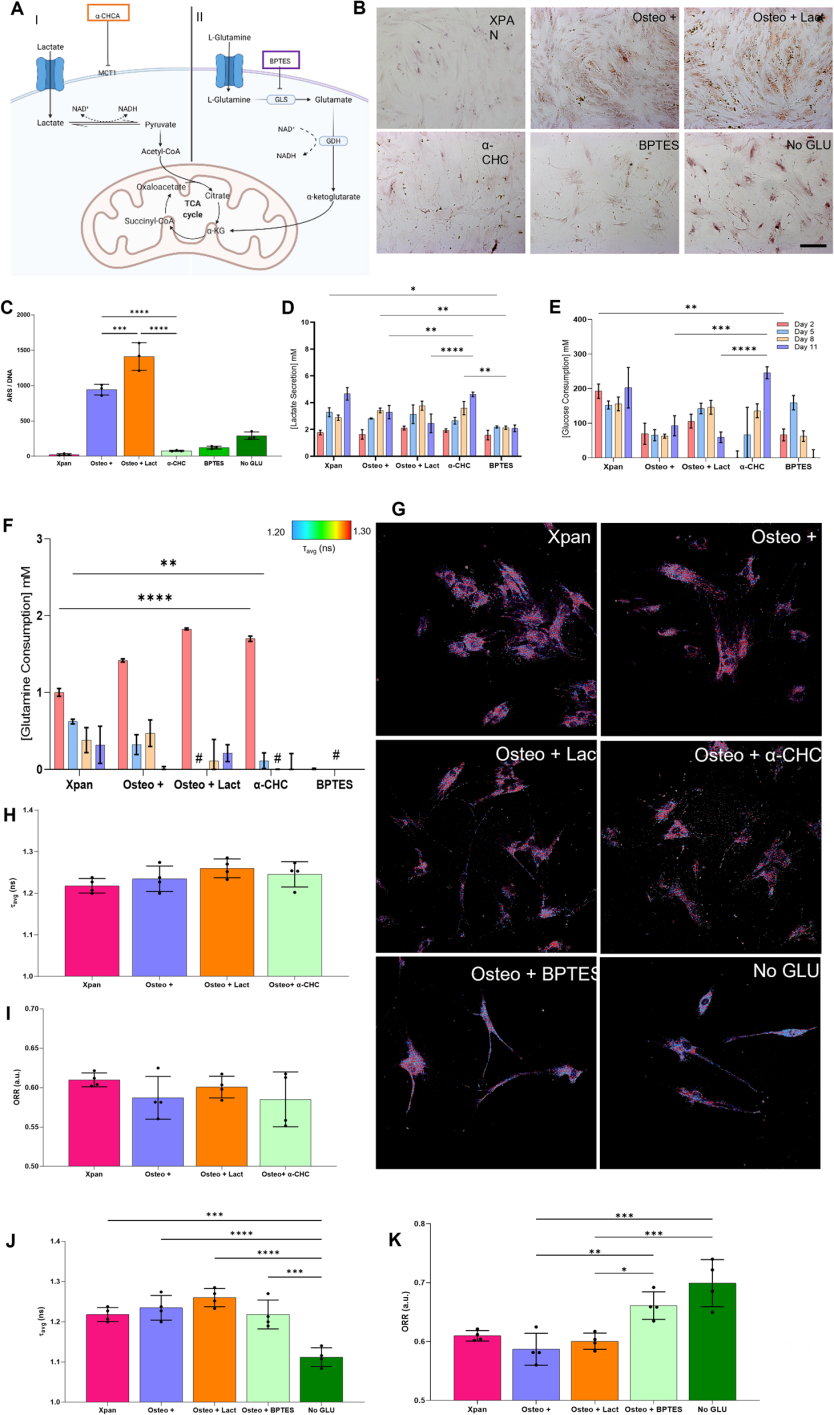
Inhibition of osteogenic differentiation using osteogenic medium supplemented with BPTES or α-CHC during 14 days of cell culture. **A** Schematic detailing the inhibition target. **B** Alizarin red staining (ARS) of hMSCs after 14 days in contact with Osteo + , Osteo + Lact, α-CHC or BPTES supplemented osteogenic medium, Osteo + medium without glutamine (No Glu) or Xpan medium. **C** Quantification of Alizarin red staining per amount of DNA after 14 days of hMSCs in various cell culture conditions. **D** hMSCs glucose consumption at day 2, 5, 8 and 11 of cell culture. **E** Lactate secretion at day 2, 5, 8 and 11 of cell culture. **F** hMSCs glutamine consumption at day 2, 5, 8 and 11 of cell culture. **G** 2P-FLIM imaging of hMSCs in different cell culture conditions **H** NAD(P)H *τ*_avg_ measurement of hMSCs after 24 h of α-CHC treatment. **I** Optical redox ratio (ORR) of hMSCs after 24 h of α-CHC treatment. **J** NAD(P)H *τ*_avg_ measurement of hMSCs after 24 h of BPTES treatment. **K** Optical redox ratio (ORR) of hMSCs after 24 h of BPTES treatment. ^#^no glutamine consumption, *p-value ≤ 0.05, **p-value ≤ 0.01, ***p-values ≤ 0.001, ****p-value ≤ 0.0001 *N* ≥ 3

Lactate supplementation of osteogenic medium promoted higher mineral deposition in hMSCs in addition to the upregulation of glutaminolysis and the reconversion from lactate to pyruvate as measured by *τ*_avg_, ORR, metabolites consumption and gene expression. To validate that osteogenic differentiation is dependent on glutaminolysis and lactate transport/metabolism, these metabolic pathways were stunted using chemical inhibitors. To inhibit glutaminolysis, BPTES, a chemical competitor of glutamate synthase enzyme, which is also the first step of the glutaminolysis pathway was used. Inhibiting this enzyme has showed to inhibit the glutaminolysis pathway [24]. Similarly, for the inhibition of lactate transport, α-CHC was used. This chemical component inhibits the uptake of lactate by blocking SLC16A1 transporter [25], whereby this transmembrane transporter has been shown to be the main entry point of lactate in hMSCs cells (Fig. 4A).

On culturing hMSCs for 14 days with Osteo + medium supplemented with α-CHC, BPTES or no glutamine added (No Glu), a reduction in alizarin red staining was observed, demonstrating lower mineral deposition (Fig. 4B). We further validated this result by calculating the amount of alizarin red stain per amount of DNA. Here, we noticed a statistically significant decrease in the alizarin red staining when hMSCs are incubated in Osteo + medium supplemented with either α-CHC or BPTES (Fig. 4C).

α-CHC treatment was found to promote glucose consumption, showing a significantly higher concentration on day 11 when compared with Osteo + medium and lactate supplemented Osteo + medium. On the other hand, BPTES treatment showed an increasing trend of glucose consumption until day 5, followed by a decrease in glucose consumption until day 11 (Fig. 4D). Similarly, α-CHC-treated hMSCs exhibited an increasing trend in lactate secretion during the culture period. On day 11, there was a statistically significant higher lactate secretion compared to Osteo + or lactate supplemented Osteo + medium. Interestingly, a stable secretion of lactate was observed during the culture period with BPTES treatment. In addition, BPTES treatment was found to promote a statistically significant decrease of lactate when compared with all the other cell culture media conditions (Fig. 4D).

α-CHC supplemented osteogenic medium was found to cause a decrease in glutamine consumption while being significantly higher when compared with Xpan at day 2. At later time points, α-CHC-treated hMSCs showed decreasing trend of glutamine consumption (Fig. 4F). As expected, BPTES supplementation of osteogenic medium had a negative impact on glutamine consumption, with hMSCs not consuming any glutamine during cell culture (Fig. 4F). 2P-FLIM of NAD(P)H was performed to assess the impact of α-CHC and BPTES on hMSCs metabolism after 24 h of treatment (Fig. 4G). α-CHC treatment was found not to affect the *τ*_avg_ and ORR measurements of hMSCs, resulting in no statistically significant change in all cell culture conditions for both *τ*_avg_ and ORR parameters (Fig. 4H, I). For BPTES, the presence or absence of glutamine resulted in distinct metabolic profiles and impacted *τ*_avg_ and ORR measurements. The removal of glutamine from osteogenic medium caused a statistically significantly decrease in the *τ*_avg_ value (0.90-fold decrease) of hMSCs compared to Xpan, Osteo + and Osteo + Lact cell culture media (Fig. 4J). BPTES treatment of osteogenic medium and the removal of glutamine from osteogenic medium resulted in an increase of 1.10-fold and 1.17-fold of ORR respectively, when compared with other cell culture media conditions (Fig. 4K).

## Discussion

This work demonstrates a potent avenue to modulate hMSCs differentiation using extracellular metabolites with a new role for the metabolite lactate uncovered that promotes increased hMSCs osteogenic differentiation via glutaminolysis. This work demonstrates not only the importance of essential metabolites in osteogenic differentiation but also showcases the highly powerful 2P-FLIM as a method to uncover, profile and validate hMSC metabolism non-invasively in real time. In this work, we monitor the metabolic profile of hMSCs during osteogenic differentiation and demonstrate that hMSCs undergoing osteogenic differentiation medium are dependent on OxPhos as a source of energy. Additionally, we also use 2P-FLIM to highlight a reliance of hMSCs on glutaminolysis and anabolic pathways as evidenced by increased glutamine consumption. In order to verify this, we supplemented osteogenic media with lactate and show that lactate supplementation of osteogenic medium can indeed promote osteogenic differentiation. To further confirm the interplay of glutaminolysis and lactate metabolism in the osteogenic differentiation of hMSCs, we inhibit these pathways using BPTES and α-CHC, respectively, and confirm the detrimental impact blocking of pathways has on the successful differentiation of hMSCs. Importantly, this study demonstrates the importance of establishing metabolic environment during cell culture or cell maturation either to drive higher differentiation rates in hMSCs or to simulate metabolic properties of native tissue. This new metabolic viewpoint in stromal cell differentiation has great potential as a tool to promote stromal cell differentiation in vitro.

hMSCs treated with osteogenic medium showed a higher *τ*_avg_, as early as day 3, reflective of a metabolic shift towards OxPhos as a consequence of a higher proportion of longer lifetime and protein-bound NAD(P)H [6] (Fig. 3F). Also, *τ*_1_ lifetimes are higher in osteogenic conditions, highlighting higher amounts of NADPH and anabolic activity [7] (Fig. 1E). Osteogenic medium induced lower ORR possibly to fuel both OxPhos and anabolic pathways (Fig. 1F). These metabolic results are in agreement with the literature; higher OxPhos and anabolic pathways are depended upon during osteogenic differentiation [14]. However, this study is the first to uncover a metabolic switch as early as day 3. UMAP and PCA distribution analysis of 2P-FLIM variables confirmed a time-dependent metabolic switch during osteogenic differentiation. Taking into account 2P-FLIM variables in the UMAP analysis, there is noticeable clustering between Xpan and Osteo + treated cells at day 7 and day 14. PCA analysis also revealed a separation at day 7 and 14 between these culture conditions. Overall, the statistical significance and the distance between PCA and UMAP clusters of both cell culture conditions increases with longer incubation periods. Mahalanobis distance, two-sample Hotelling’s T2 test, *F*-value, *F*-critical and *p*-values were calculated to ensure statistical significance between PCA cluster groups (Additional file 1: Table 2). The clear and statistically valid segregation of cell culture and hMSCs profiles demonstrate the possibility to use PCA and UMAP not only as data visualization tools but to be used as classification tools with 2P-FLIM photonic measurements as the source of this data. The trends observed with 2P-FLIM and extracellular metabolite quantification confirm this metabolic switch towards OxPhos by the reduction in the glucose consumption while showing a higher dependence of glutaminolysis at earlier time points. The metabolic switch towards increased OxPhos dependence is a known feature of differentiated cells and has been documented during osteogenic differentiation [26]. Interestingly, glutamine has been shown to be a critical metabolite to achieve osteogenic differentiation [5]. One study by Yu et al. demonstrated that glutamine is an important metabolite to achieve osteogenic differentiation by its anaplerotic role in feeding the Krebs cycle by proxy of alpha-ketoglutarate. In addition, this study demonstrated that glutamine removal impacts osteogenic differentiation by a decrease in the alizarin red staining [5]. Notably, Gayatri et al. [27] demonstrated that increasing glutamine concentrations tenfold supresses osteogenic differentiation of human and murine MSCs. Therefore, to promote glutaminolysis and anaplerotic pathways and consequently promote earlier osteogenesis, the glutaminolysis pathway rate needs to be increased without necessarily increasing extracellular concentrations of glutamine.

To achieve a higher rate of glutaminolysis, we chose to upregulate the glutaminolysis pathway by modifying the Osteo + cell culture medium formulation. Pérez-Escuredo et al. demonstrated an upregulation of glutamine uptake and glutaminolysis promoted by increased lactate concentration on oxidative tumour cells [22]. Inspired by this, we increased exogenous lactate levels in the Osteo + medium to promote higher glutaminolysis, osteogenic differentiation in hMSCs and mineral deposition. To account for pH changes due to increased lactate concentration, exogenous lactate in the form of sodium lactate was used to stabilize pH values [22]. Lactate supplementation of osteogenic media yielded a higher mineral deposition and osteogenic differentiation (Fig. 3A, B, C). Here, increased gene expression of ALPL, SOX9, SPP1 and RUNX2 are upregulated markers of osteogenesis in hMSCs and follow the trends observed in literature [28]. Using 2P-FLIM, we observed a shift in metabolism when supplementing osteogenic medium with lactate (Fig. 3E). A lower *τ*_avg_ shows an increase in free NAD(P)H fraction in the cytoplasm concomitant with a reduction of protein-bound NADH. This shift in NAD(P) fraction is associated with higher metabolic dependence on glycolysis (Fig. 3F). Simultaneously, there was a decrease in ORR associated with OxPhos and anaplerotic metabolic pathways such as glutaminolysis (Fig. 3G). UMAP and PCA distribution analysis of 2P-FLIM data revealed a segregation between the hMSCs treated with Osteo + SL, Osteo + and Xpan medium (Fig. 4D, E). This shows that the three cell culture formulations promote distinct metabolic signatures in hMSCs. Metabolic gene expression revealed for most genes, higher expression levels when hMSCs are cultured for 14 days in osteogenic lactate supplemented cell culture medium (Fig. 3). These glutaminolysis genes are responsible for the conversion of glutamine to glutamate and the conversion of glutamate to alpha-ketoglutarate in the cytoplasm and the mitochondrial matrix [29]. These poly-hydrolases, which are located within the endoplasmic reticulum, are activated by the presence of alpha-ketoglutarate and are crucial for the formation of the collagen triple helix. The incomplete hydroxylation of proline residues impacts collagen triple helix formation reducing collagen secretion to the cytoplasm and lastly reducing extracellular secretion of collagen [30]. The ability to secrete high amounts of extracellular matrix such as collagen I is a known feature of osteoblasts and important during bone formation [31]. Lactate metabolic genes are also highly expressed in lactate supplemented osteogenic medium when compared with non-supplemented osteogenic medium. SL16A1 in particular has been showed to express MCT1 one of the main membrane transporters of lactate in hMSCs [32]. LDH is also highly expressed in all isoforms when treating hMSCs with Osteo + cell culture medium. LDH is responsible for conversion of pyruvate to lactate and is capable of performing the reverse enzymatic reaction [33]. The upregulation of LDH gene expression without an increase in extracellular lactate concentrations demonstrates that LDH has a higher ratio of reverse enzymatic reaction compared with the forward reaction. Glutaminolysis and lactate gene expression clarifies that exogenous lactate supplementation of Xpan medium promotes upregulation of glutaminolysis while increasing lactate uptake and reconversion to pyruvate in hMSCs. PCA metabolic gene expression analysis revealed a statistically significant segregation between all cell culture conditions (Fig. 2J). This confirms that lactate supplementation of osteogenic medium is having a direct impact on the metabolism of hMSCs. hMSCs when treated with Osteo + Lact medium have higher level of glutaminolysis rate, higher uptake of lactate, higher rates of lactate conversion to pyruvate and higher secretion levels of collagen. These metabolic shifts can be therefore responsible for higher levels of osteogenic differentiation. A recent study supplementing HepG2 cultures with L-lactate and D-lactate supports the findings here, whereby lactate supplementation increased pyruvate utilization [34].

After showing the significance of glutaminolysis and lactate during osteogenic differentiation, we decided to block glutaminolysis using BPTES or removing glutamine (No Glu) from the osteogenic medium, as well as blocking the uptake of lactate using α-CHC (Fig. 4A). BPTES directly impacts the GLS enzyme [24] while α-CHC inhibits the SLC16A1 transporter, responsible for lactate uptake [35]. Alizarin red staining and extracellular metabolic analysis show that inhibiting lactate uptake and glutaminolysis negatively impacts osteogenic differentiation. 2P-FLIM NAD(P)H measurements of the metabolic profile of hMSCs after 24 h treatment demonstrated that α-CHC does not impact either *τ*_avg_ or ORR redox ratio at earlier treatments times. Since α-CHC directly inhibits a transmembrane transporter responsible for lactate uptake, the impact on metabolism might not be pronounced in short culture times probably due to the pyruvate available in the medium which can evade lactate uptake to be converted back to pyruvate while generating NADH. However, BPTES treatment or removal of glutamine significantly impacted hMSCs metabolism after 24 h. Removal of glutamine produced a statistically significant decrease of *τ*_avg_ possibly generated by the cell compensating the absence of an essential metabolite by increasing glycolysis to fuel the Krebs cycle for cellular differentiation. ORR values have a statically significant increase when hMSCs are cultured in the presence of BPTES and no glutamine osteogenic medium. Increased ORR due to BPTES or removal of glutamine results from a reduction in the NADH availability due to not being regenerated during glutamate conversion to alpha-ketoglutarate [20]. Lactate was added to osteogenic medium in the presence of BPTES and α-CHC in order to complement glutaminolysis and lactate transport inhibition studies in hMSCs. Lactate supplementation of osteogenic medium with BPTES was not able to rescue osteogenic differentiation. In addition, lactate supplementation of osteogenic medium with α-CHC was also not able to promote osteogenic differentiation (Additional file 1: Fig. 2). This demonstrates that osteogenic differentiation supplemented with lactate impacts directly both metabolic pathways: glutaminolysis and lactate uptake.

The culmination of these results validates the role of glutaminolysis in the osteogenic differentiation of hMSCs observed by Yu et al. in which blocking glutaminolysis with BPTES negatively impacted osteogenic differentiation [5]. Our results underline that both metabolic pathways are essential for osteogenic differentiation and glutaminolysis with the direct and indirect role of lactate in the metabolism of hMSCs. In this study, a new role for lactate in osteogenic differentiation of hMSCs is uncovered. Here, lactate can act indirectly on osteogenic differentiation to upregulate glutaminolysis and directly by being used as metabolic fuel to regenerate pyruvate. Only recently, lactate has started to be understood as a fuel source and a distinct signalling molecule as opposed to a metabolic by-product. It is emerging as an interesting metabolite, for instance documented to contribute to reduce inflammatory cell signalling [36]. Lactate’s role in osteogenesis has polarizing views. Generally, this is because the effects of lactate are largely dependent on the concentration as well as its subcellular localization. Earlier studies have reported the addition of lactate in the form of lactic acid as having an inhibitory effect on osteogenesis [37], with others attributing l-lactic acid and d,l-lactic acid (as a model of degradation from poly(lactic acid) biomaterials) impacting osteogenesis primarily due to the alteration of media pH [38]. Conversely, Wu et al. [39] have reported improved osteoblast differentiation through the addition of l-(+)lactic acid and linking to a role in enhancing parathyroid hormone via GPR91-PKC-Akt signalling. In a separate study, the same group also reported lactic acid induction of osteoblast differentiation via stabilization of HIF1α, a study which has complimentary findings with the study of this 2P-FLIM paper [40]. However, the focus of Wu’s study was specifically on aerobic glycolysis, but still underlines a potent impact of interrogating metabolism using metabolites to drive cell differentiation. Recently, Luo et al. have documented an inhibitory effect of lactate on osteogenic differentiation of human periodontal ligament stem cells via autophagy. Their interpretation is that lactate reduced the expression of ALP via GPR81-Gα subunit signalling and inhibited mineralized nodule formation through the MCT1-mTOR signally pathway [41]. Another more recent study flips back to the other side of the lactate debate, showing that in vitro cyclical mechanical stretch induced ALP activity and mineralized nodules in alveolar bone marrow mesenchymal cells (ABMMCs) which can be inhibited by GNE-140 (a specific lactate dehydrogenase A (LDHA)) inhibitor to inhibit the production of lactate, therefore linking lactate to osteogenic differentiation. Benchmarking against several of these studies finds agreeing and conflicting findings and may be dependent upon the variability in sensitivities, concentrations of lactate tested and response of cells to cultivation conditions. The role and impact of lactate still merit further study and should be carefully assessed pertaining to the cell type and state being studies, microenvironmental factors such as pH, osmolarity and the availability of energy sources (oxygen tension, glucose/sugar, fatty acids, amino acids) and the concentration of lactate being used. The concentration used in this study is 7.5 mM which is in close range of that of the studies being mentioned (typically 5–10 mM), and it is the first time that a metabolic link between lactate supplementation and glutaminolysis, by proxy of 2P-FLIM, is established. One this is for sure though, this once regarded waste product of metabolism should be considered as a metabolite which can moonlight as a potent contributor of cell differentiation.

## Conclusions

In conclusion, we show that osteogenic differentiation is a phenotypic and metabolic process that occurs in a time-dependent manner that is reliant on glutaminolysis and lactate. The inhibition of these pathways using BPTES and α-CHC and withdrawal of glutamine negatively impacts osteogenic differentiation. The supplementation of osteogenic medium with lactate promoted higher osteogenic differentiation, glutaminolysis, lactate metabolism and endpoint poly-hydrolyses gene expression. 2P-FLIM enabled monitoring of these effects non-invasively over time to infer glycolysis and information on anabolic pathways such as glutaminolysis. This non-invasive mode of characterization unveiled metabolic behaviours that steered us to focus on glutaminolysis and drive it further using sodium lactate. Furthermore, we were able to use the 2P-FLIM photonic data to create UMPA- and PCA-based signatures to cluster hMSC differentiation. Potentially, the use of sodium lactate (or some alternative form of lactate) as a coating or as a biocompatible material in the form of lactic acid (PLA) can promote further stromal cell differentiation at an implantation site. The metabolic role of lactate as fuel and signalling molecule is still a new approach to a metabolite that is traditionally considered as a metabolic by-product and established as a cellular metabolism endpoint. Our research and that of others is now shining new light on the impact of exogenous lactate on cellular function.

### Supplementary Information

**Additional file 1: Table S1.** Gene symbol, name, accession number, unique assay ID and amplicon length of genes used for qPCR. **Fig. S1.** Alizarin red staining of hMSCs after 21 days of incubation in either Xpan or Osteo+ cell culture media. **Table S2.** Mahalanobis distance, Hotelling’s T2 stats, F-value, critical F-Values, P-value and significance results of PCA statistical significance analysis. **Fig. S2.** Supplementation of Osteo+ cell culture medium with lactate (7.5 mM) and metabolic inhibitors. **A** Alizarin red staining of hMSCs after 14 days of cell culture in several cell culture medium formulations. **B** Alizarin red quantification per DNA of hMSCs after 14 days of cell culture.

## Data Availability

Data and source files are available within reasonable request.

## Abbreviations

*α*_*n*_: Fraction of fluorescence lifetime
ANOVA: Analysis of variance
ETC: Electron transport chain
FAD: Flavin adenine dinucleotide
FBS: Foetal bovine serum
FCCP: Carbonyl cyanide-*p*-trifluoromethoxyphenylhydrazone
FLIM: Fluorescence lifetime imaging microscopy
hMSCs: Human mesenchymal stem cells
Laser: Light amplification by stimulated emission of radiation
MHz: Megahertz
NAD(P)H: NADH + NADPH
NADH: Nicotinamide adeninine dinucletodide
NADPH: Nicotinamide adenine phosphate dinucleotide
nm: Nanometre
ns: Nanosecond
ORR: Optical redox ratio
Osteo +: Osteogenic cell culture medium
Osteo + Lact: Osteogenic medium supplemented with lactate
OxPhos: Oxidative phosphorylation
PCA: Principal component analysis
PCR: Polymerase chain reaction
Pen/Strep: Penicillin and streptomycin
qPCR: Quantitative PCR
ROS: Reactive oxygen species
RT-PCR: Real-time PCR
TCA: Tricarboxylic acid cycle
TCSPC: Time-correlated single-photon counting
t-SNE: t-distributed stochastic neighbour embedding
μm: Micrometre
UMAP: Uniform manifold approximation and projection
χ^2^: Chi-squared
Xpan: Expansion/growth cell culture medium
*λ* (nm): Wavelength
*τ*_*n*_: Fluorescence lifetime

## Declarations

**Ethical approval and consent to participate.**

Not applicable.
