# Supplementary material for: 2P-FLIM unveils time-dependent metabolic shifts during osteogenic differentiation with a key role of lactate to fuel osteogenesis via glutaminolysis identified
Source: Stem Cell Res Ther. 2023 Dec 12;14:364. doi: 10.1186/s13287-023-03606-y (PMC10717614; doi:10.1186/s13287-023-03606-y)
Supplement: Supplementary file 1 — Additional file 1: Table S1. Gene symbol, name, accession number, unique assay ID and amplicon length of genes used for qPCR. Fig. S1. Alizarin red staining of hMSCs after 21 days of incubation in either Xpan or Osteo+ cell culture media. Table S2. Mahalanobis distance, Hotelling’s T2 stats, F-value, critical F-Values, P-value and significance results of PCA statistical significance analysis. Fig. S2. Supplementation of Osteo+ cell culture medium with lactate (7.5 mM) and metabolic inhibitors. A Alizarin red staining of hMSCs after 14 days of cell culture in several cell culture medium formulations. B Alizarin red quantification per DNA of hMSCs after 14 days of cell culture. [file 13287_2023_3606_MOESM1_ESM.docx]

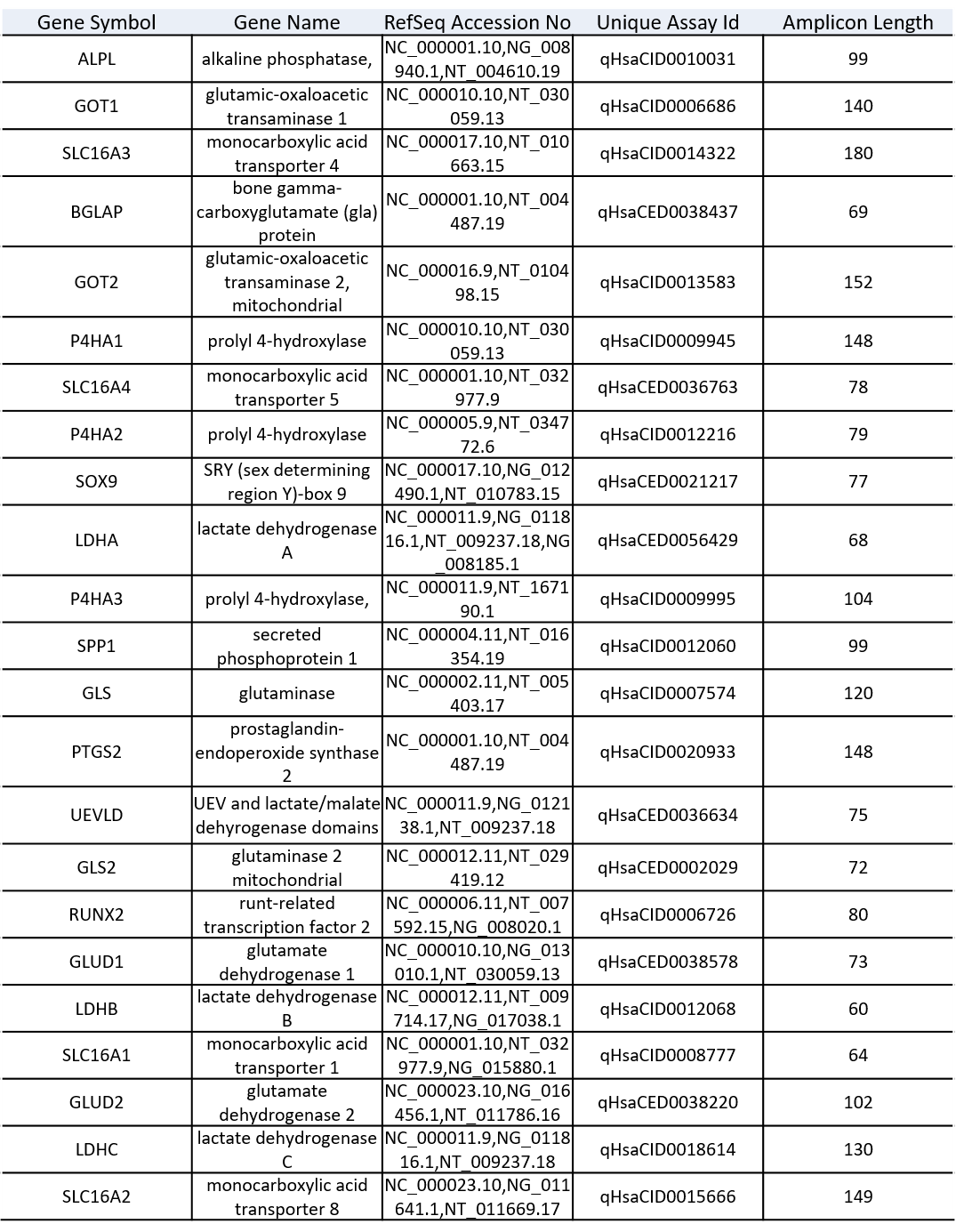


Supplemental Table 1 – Gene symbol, name, accession number, unique assay ID and amplicon length of genes used for qPCR

Table Appendix 2 – Mahalanobis distance, Hotelling T^2^ stats, F-value, Critical F-Values, P-value and significance results of PCA statistical significance analysisTable Appendix 3 – Gene symbol, name, accession number, unique assay ID and amplicon length of genes used for qPCR

Supplemental Figure 1 - Alizarin red staining of hMSCs after 21 days of incubation in either Xpan or Osteo+ cell culture media.


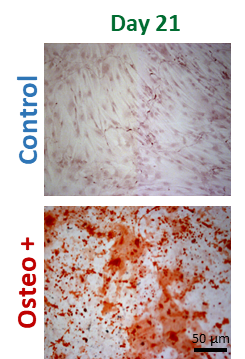


Supplemental Table 2 – Mahalanobis distance, Hotelling T^2^ stats, F-value, Critical F-Values, P-value and significance results of PCA statistical significance analysis

|  | Mahalanobis Distance | Two-Sample T2 Stats | F-Value | Critical F-Value | P-value | Significance |
| --- | --- | --- | --- | --- | --- | --- |
| Osteo vs Xpan | 4.121 | 25.478 | 9.554 | 9.552 | 0.025 | YES |
|  |  |  |  |  |  |  |
| Figure 4.3 |  |  |  |  |  |  |
| Osteo vs Xpan (D0) | 1.165 | 8.151 | 3.89 | 3.467 | 0.2063 | NO |
| Osteo vs Xpan (D3) | 1.986 | 23.665 | 11.295 | 3.467 | 0.105 | NO |
| Osteo vs Xpan (D7) | 3.223 | 62.314 | 29.748 | 3.467 | 0.044 | YES |
| Osteo vs Xpan (D14) | 5.32 | 169.823 | 81.052 | 3.467 | 0.012 | YES |
|  |  |  |  |  |  |  |
| Figure 4.5 |  |  |  |  |  |  |
| Osteo vs Xpan | 3.105 | 49.152 | 11.264 | 9.552 | 0.048 | YES |
| Xpan vs Lact | 11.06 | 183.472 | 68.802 | 9.552 | 0.0005 | YES |
| Lact vs Osteo | 6.4905 | 63.19 | 23.696 | 9.552 | 0.006 | YES |
|  |  |  |  |  |  |  |
| Figure 4.6 |  |  |  |  |  |  |
| Xpan vs Osteo | 5.569 | 139.584 | 65.431 | 3.682 | 0.01 | YES |
| Osteo vs Lact | 3.127 | 43.992 | 20.621 | 3.682 | 0.0472 | YES |
| Lact vs Xpan | 3.888 | 68.019 | 31.884 | 3.682 | 0.0289 | YES |
|  |  |  |  |  |  |  |
| Figure 4.8 |  |  |  |  |  |  |
| Osteo vs Xpan | 7.513 | 84.666 | 31.75 | 9.552 | 0.0034 | YES |
| Xpan vs Lact | 5.232 | 41.059 | 15.397 | 9.552 | 0.0127 | YES |
| Lact vs Osteo | 4.942 | 36.641 | 13.74 | 9.552 | 0.0151 | YES |


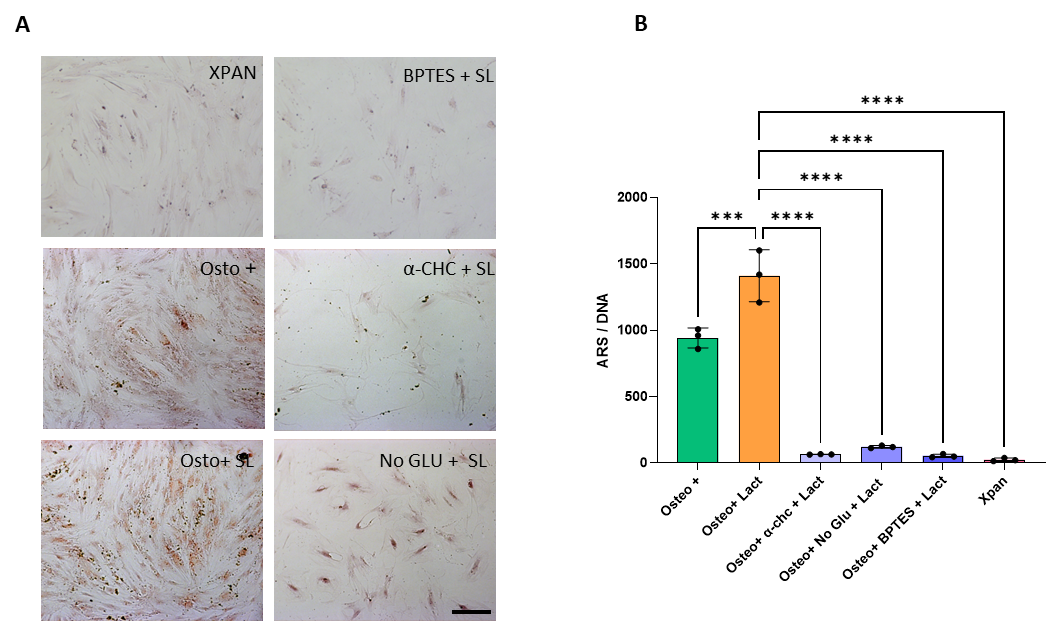


Supplemental Figure 2 – Supplementation of Osteo + cell culture medium with lactate (7.5 mM) and metabolic inhibitors. (A) Alizarin red staining of hMSCs after 14 days of cell culture in several cell culture medium formulations. (B) Alizarin red quantification per DNA of hMSCs after 14 days of cell culture
